# Supplementary material for: Synthesis and Modeling of Ezetimibe Analogues
Source: Molecules. 2021 May 22;26(11):3107. doi: 10.3390/molecules26113107 (PMC8196997; doi:10.3390/molecules26113107)
Supplement: Supplementary file 1 [file molecules-26-03107-s001.zip › molecules-1186173-supplementary.pdf]

Materials and Methods

The reactions performed with air sensitive reagents were conducted under dry argon. The solvents were previously distilled under nitrogen over calcium hydride or sodium filaments. Melting points were determined in a Koffler apparatus and are not corrected. NMR spectra were recorded Both proton NMR (^{1}H NMR) and carbon NMR (^{13}C NMR) were recorded in deuterated solvents, on spectrometers working on 200 MHz or 400 MHZ for proton and 50 MHz or 100 MHz for carbon. Data shown below is represented as follows: chemical shift, multiplicity, coupling constant, integral and assignment. Chemical shifts are reported in ppm with respect to residual solvent protons, coupling constants ($J_{X\text{-}X'}$) are reported in Hz. Mass spectra were taken in Mass spectra was recorded using APCI (Atmospheric Pressure Chemical Ionization). Rotatory power data was recorded using $CHCl_3$ as solvent and sodium D line as polarized light ray: IR data was recorded using liquid IR spectrometer and NaCl crystal as supporting material. Silica column chromatography was performed using silica gel 60A (0.060-0.200 mm).

1. Baylis-Hillman reaction between *tert*-butyl acrylate and *p*-methoxybenzaldehyde.

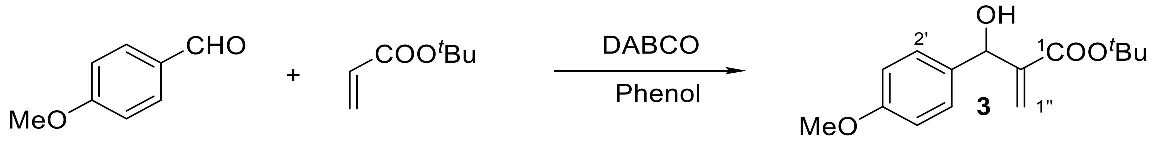

Over 1.01 g (7.50 mmol) of *p*-anisaldehyde (CAS: 123-11-5, light yellow liquid), 3.40 g of DABCO (CAS: 280-57-9, white powder) (7.50 mmol), 1.45 g (7.50 mmol) of phenol (CAS: 108-95-2, colourless crystalline solid) and 4.0 mL (46.3 mmol) of *tert*-butyl acrylate (CAS: 1663-39-4, colourless liquid) were added under Ar atmosphere. After 29 days, the reaction crude was dissolved in EtOAc and washed with 1 M HCl, H_2O, 10% aqueous $NaHCO_3$, H_2O and saturated aqueous NaCl. Then, 10 mL of a 40% $NaHSO_3$ aqueous solution were added to the organic phase and then stirred for 2 hours. The phases were decanted and the organic phase was washed with H_2O three times and saturated aqueous NaCl and dried over anhydrous Na_2SO_4, filtered and the solvent was evaporated, obtaining 1.77 g (6.20 mmoles), 82.0% of **3**.

3: *tert*-Butyl 3-(4-methoxyphenyl)-3-hydroxy-2-methylene-propanoate.

^{1}H NMR (200 MHz, $CDCl_3$) δ (ppm): 7.20 (d, J = 8.7 Hz, 2H, H-2'), 6.79 (d, J = 8.7 Hz, 2H, H-3'), 6.17 (s, 1H, H-1''B), 5.73 (s, 1H, H-1''A), 5.38 (s, 1H, H-3), 3.71 (s, 3H, -OMe), 1.33 (s, 9H, -*t*Bu).

^{13}C NMR (50 MHz, $CDCl_3$) δ (ppm): 165.83 (C, COO*t*Bu), 159.23 (C, C-4'), 144.14 (C, C-2), 134.23 (C, C-1'), 128.20 (CH, C-2'), 124.43 (CH2, C-1''), 113.82 (CH, C-3'), 81.47 (C, *t*Bu), 72.80 (CH, C-3), 55.34 (CH_3, OMe), 28.10 (CH_3, $C(CH_3)_3$).

IRυ_{max} (film) (cm^{-1}) 831, 851, 1034, 1150, 1173, 1252, 1304, 1369, 1393, 1458, 1512, 1605, 1701, 2978.

E.M.A.R., FAB, calculated for $C_{15}H_{20}O_4$ [M + Na]: 287.1253. Found: 287.1256 Δ = 0.76 ppm.

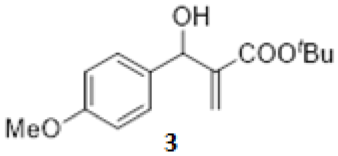
OH
COO^{t}Bu
MeO

3

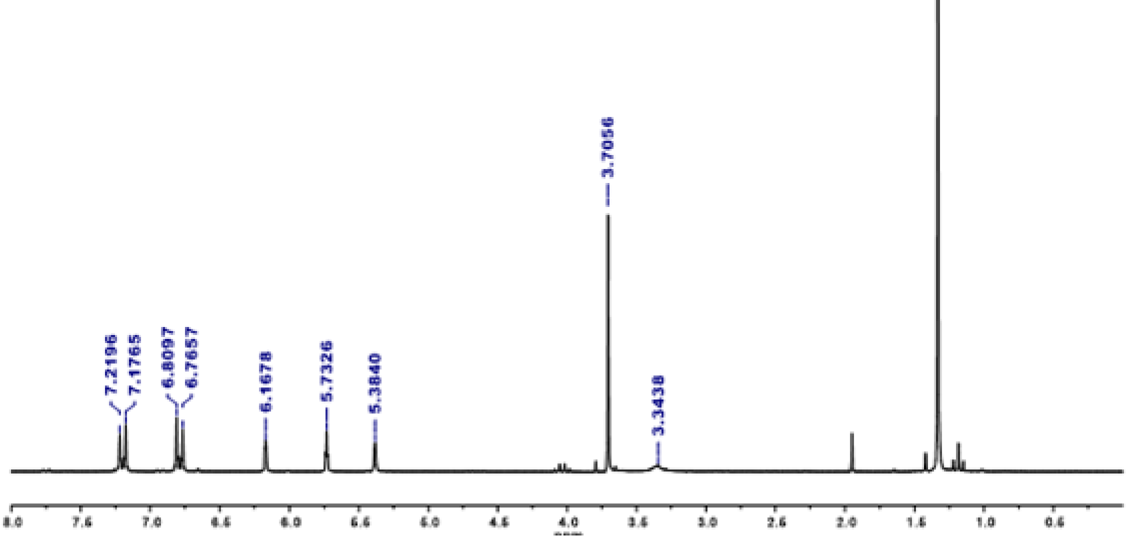
7.2196
7.1765
6.8097
6.7657
6.1678
5.7326
5.3840
3.7056
3.3438
1.3320

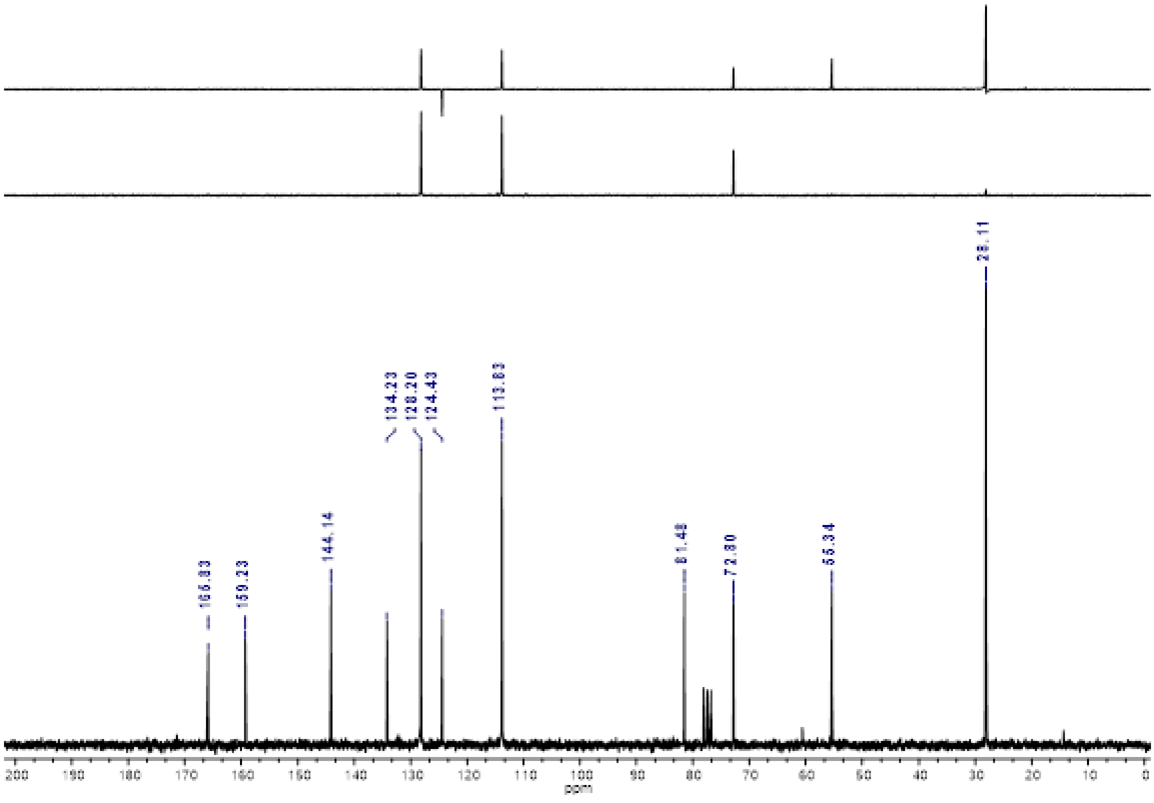
166.83
159.23
144.14
134.23
128.20
124.43
113.83
81.48
72.80
55.34
28.11
ppm

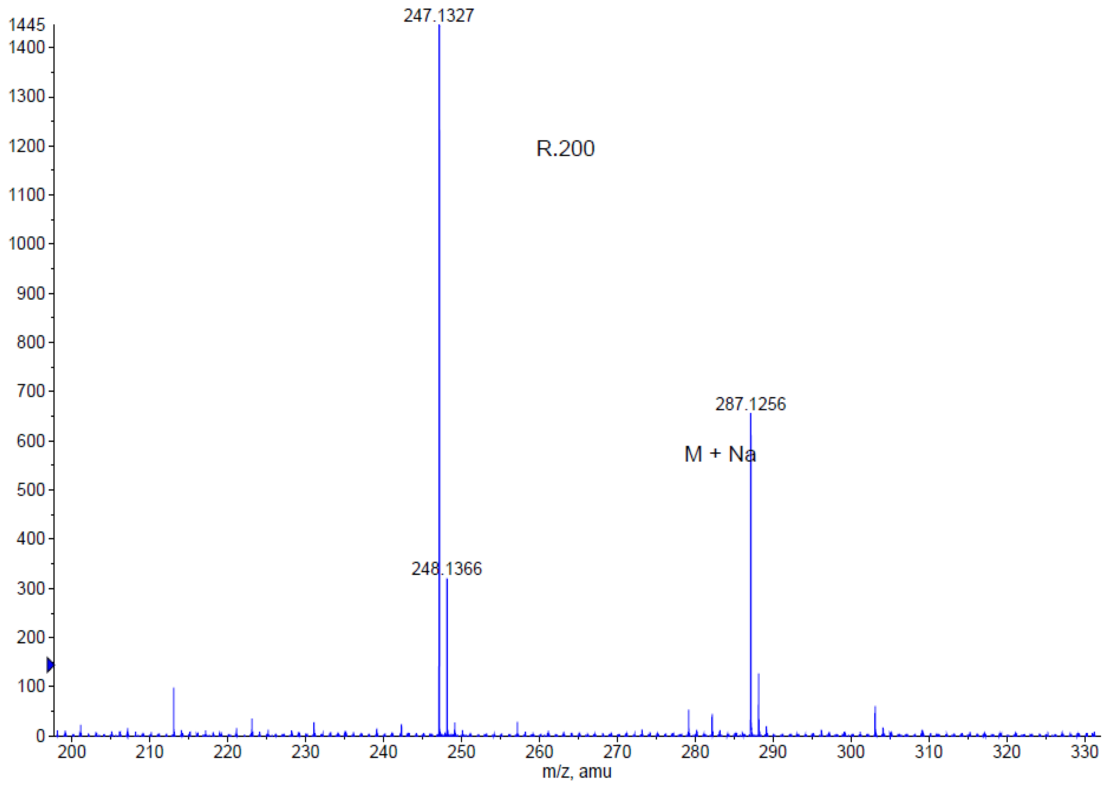
247.1327
R.200
287.1256
M + Na
248.1366
m/z, amu

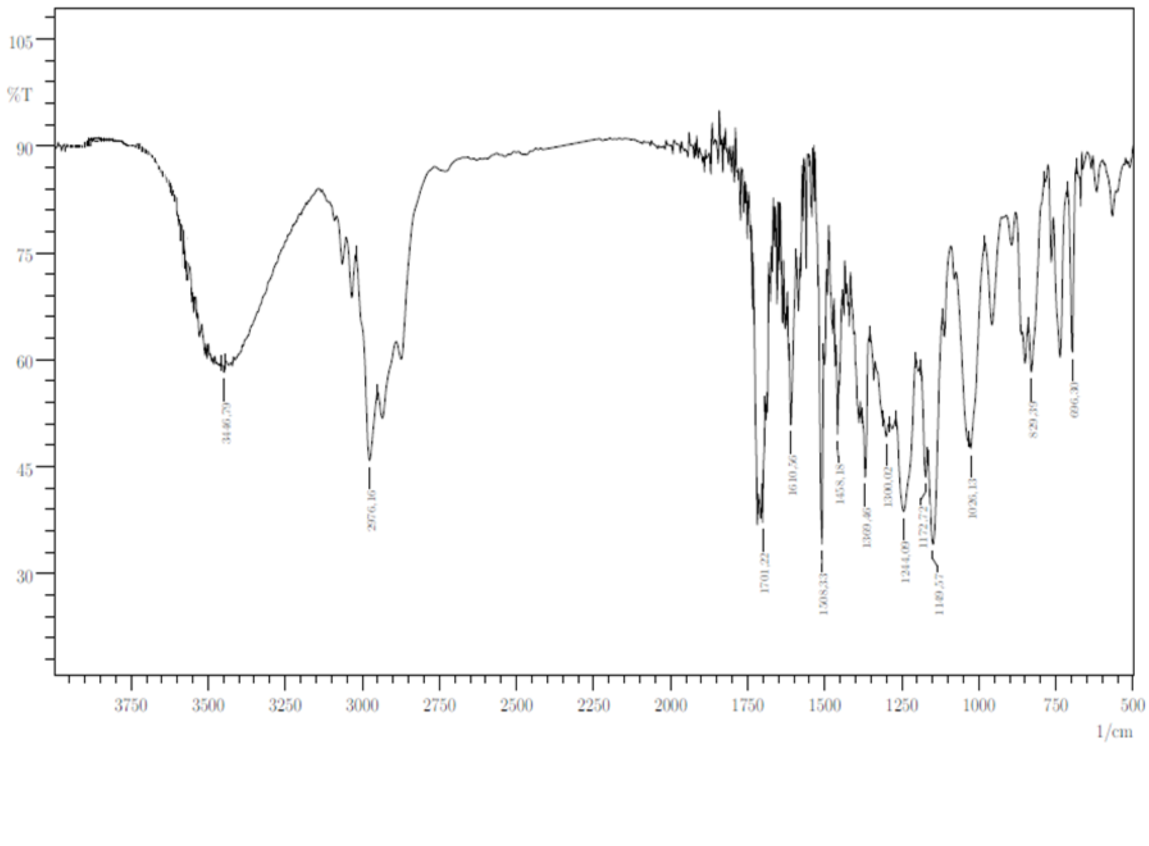
%T
3446,79
2976,16
1701,22
1610,56
1508,33
1458,18
1369,46
1300,02
1244,09
1172,72
1149,57
1026,13
829,39
696,30
1/cm

2. Acetylation of Baylis-Hillman adduct 3.

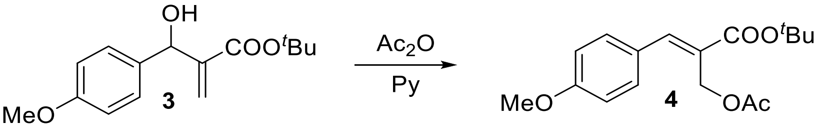

4 mL (49.50 mmol) of pyridine (CAS: 110-86-1, colourless liquid) and 3 mL (31.50 mmol) of acetic anhydride (CAS: 108-24-7, colourless liquid) were added to 1.56 g (5.92 mmol) of **3** under Ar atmosphere and at 0 ºC. The ice bath was then removed and the mixture was allowed to stir at room temperature. After 72 hours, the reaction crude was hydrolysed with ice. It was extracted with EtOAc and washed with 1 M HCl, H_2O, 5% aqueous $NaHCO_3$, H_2O and saturated aqueous NaCl. The organic phase was dried over anhydrous Na_2SO_4. The solvent was evaporated obtaining 1,68 g of crude reaction that were chromatographed on silica "flash". The desired product was eluted with hexane/AcOEt 95: 5, 1.58 g of **4** (87%) were obtained.

4: *tert*-butyl (*E*)-2-(acetoxymethyl)-3-(4-methoxyphenyl)acrylate.

^{1}H NMR (200 MHz, $CDCl_3$) δ (ppm): 7.33(d, J = 8.8 Hz, 2H, H-2'), 6.91 (d, J = 8.8 Hz, 2H, H-3'), 7.82 (s, 1H, H-3), 4.94 (s, 2H, H-1''), 3.82 (s, 3H, -OMe), 2.10 (s, 3H, -OAc), 1.53 (s, 9H, COO*t*Bu).

^{13}C NMR (50 MHz, $CDCl_3$) δ (ppm): 171.62 (C, OCOMe), 166.52 (C, COO*t*Bu), 160.82 (C, C-4'), 144.25 (C, C-2'), 131.54 (C, C-3), 127.21 (CH, C-1'), 126.42 (C, C-2'), 114.39 (CH, C-3'), 81.29 (C, $C(CH_3)_3$), 59.87 (CH_2, C1''), 55.55 (CH_3, -OMe), 28.38 (CH_3, $C(CH_3)_3$), 21.24 (CH_3, OCOMe).

IRυ_{max} (film) (cm^{-1}): 829, 1028, 1150, 1175, 1202, 1231, 1252, 1288, 1395, 1512, 1611, 1699, 1719, 2936, 2978.

E.M.AR., FAB, calcd for $C_{17}H_{22}O_5$ [M + Na]: 329.1359. Found: 329.1360. Δ = -0.14 ppm.

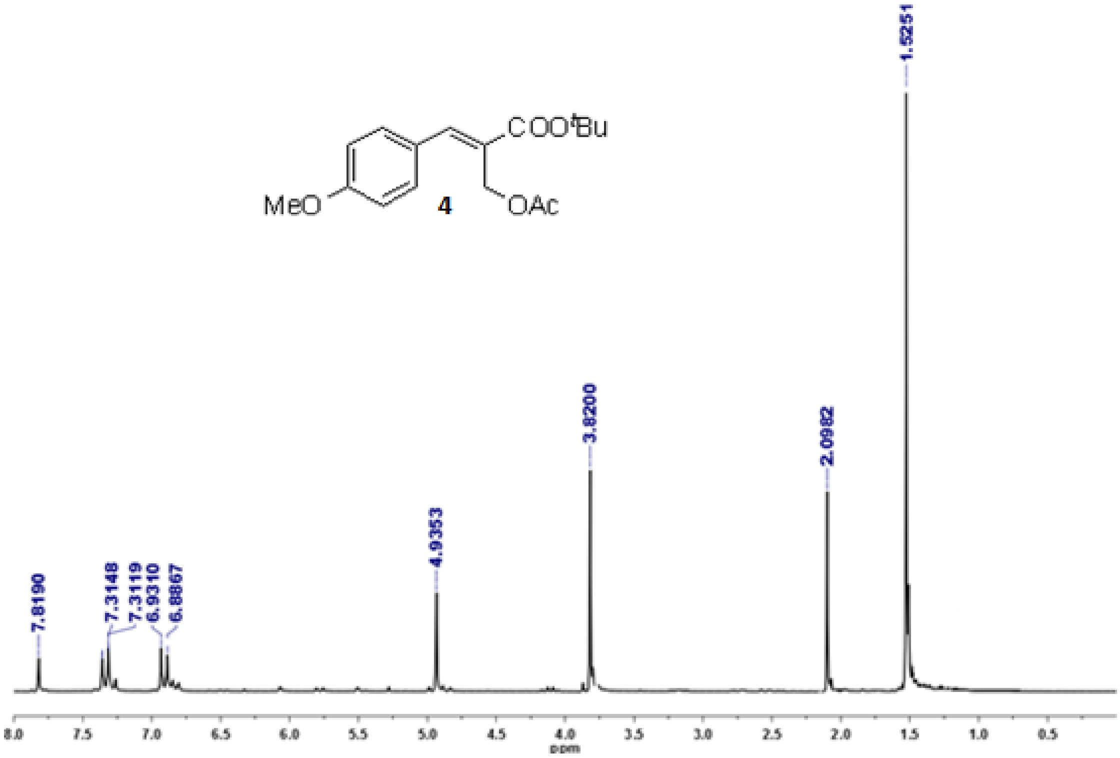

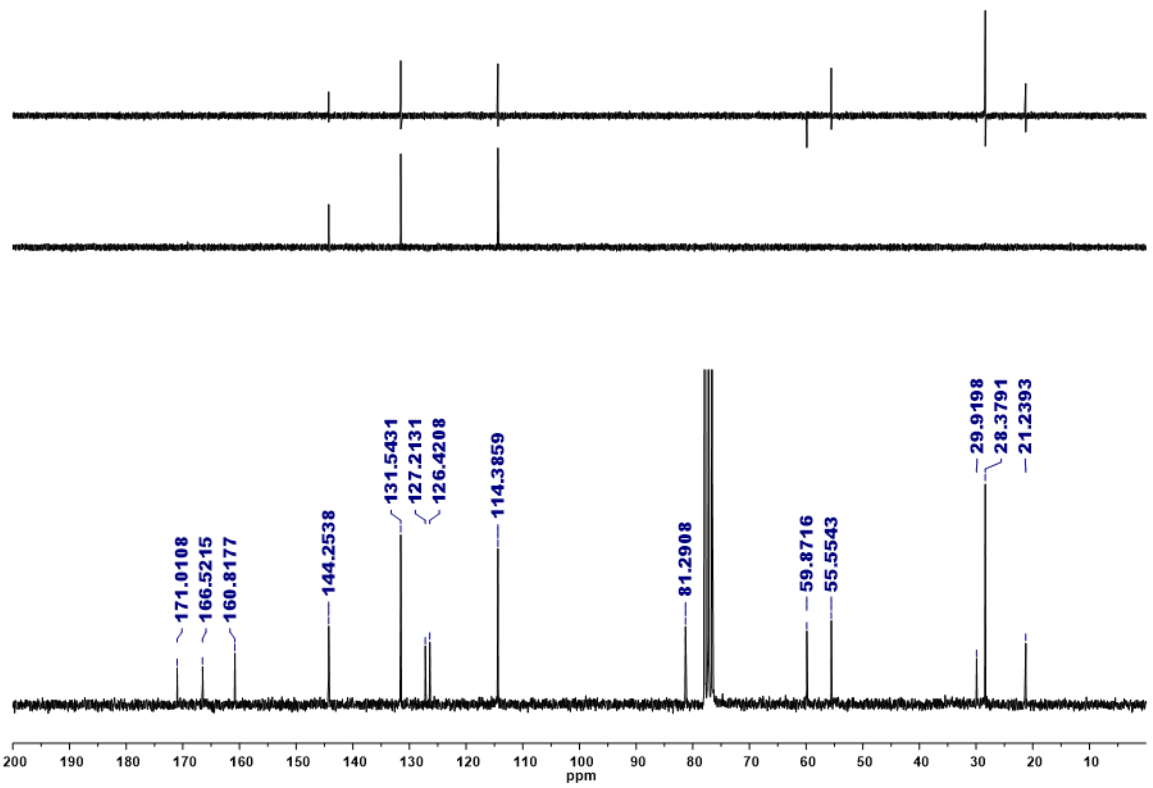
171.0108
166.5215
160.8177
144.2538
131.5431
127.2131
126.4208
114.3859
81.2908
59.8716
55.5543
29.9198
28.3791
21.2393
200 190 180 170 160 150 140 130 120 110 100 90 80 70 60 50 40 30 20 10
ppm

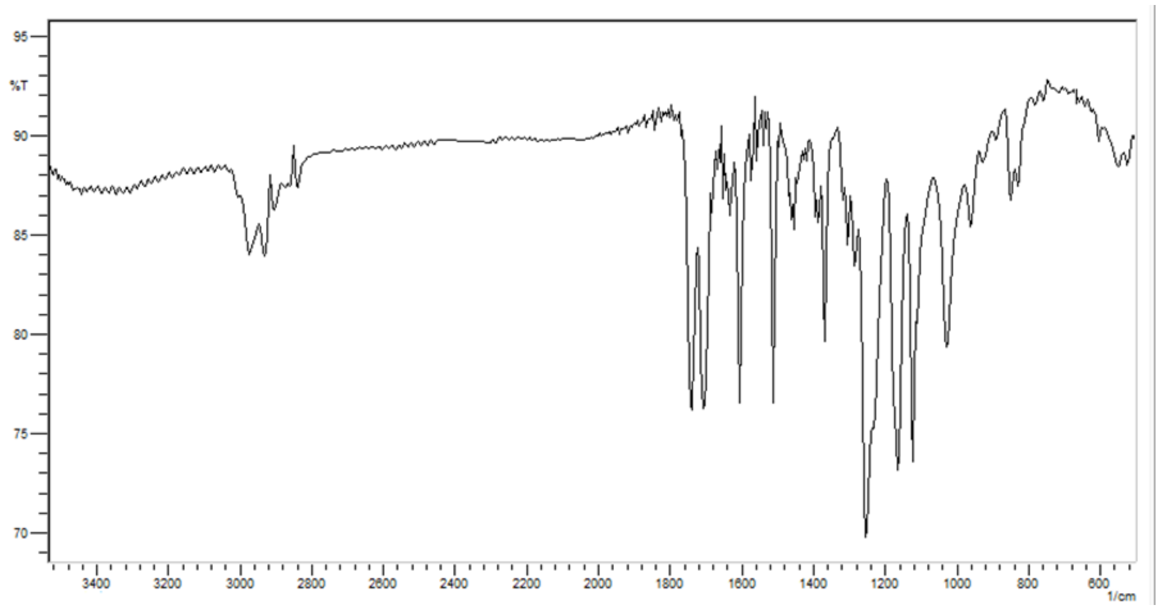
95
%T
90
85
80
75
70
3400 3200 3000 2800 2600 2400 2200 2000 1800 1600 1400 1200 1000 800 600
1/cm

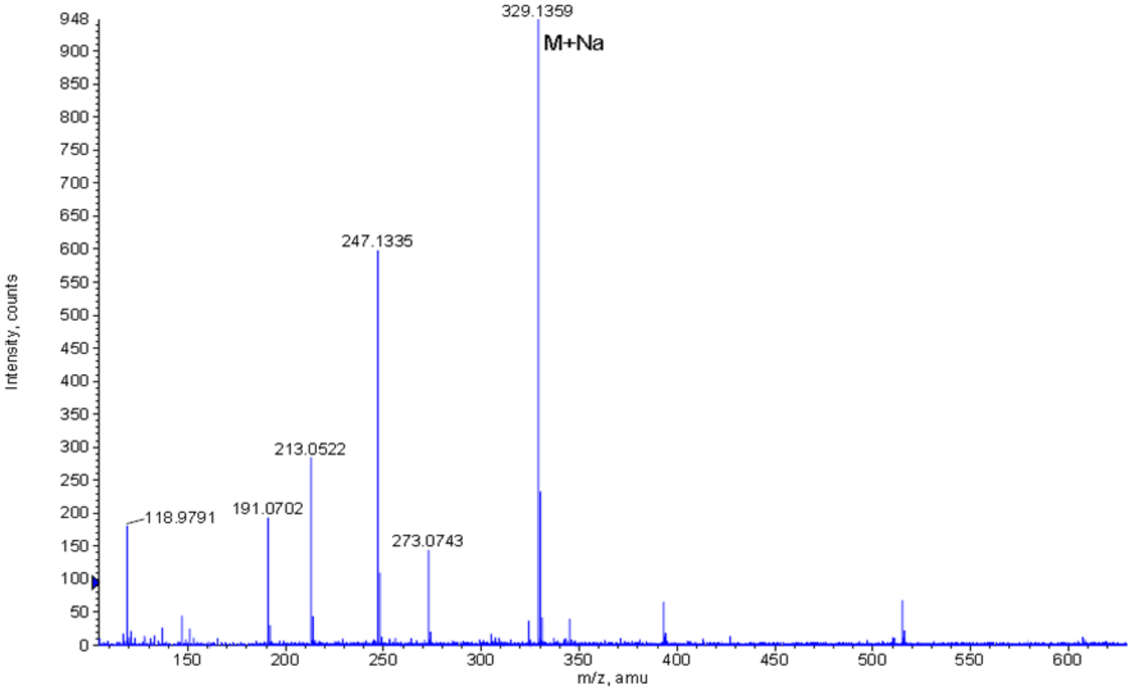

3. Addition of chiral amide (*R*)-2 to 4.

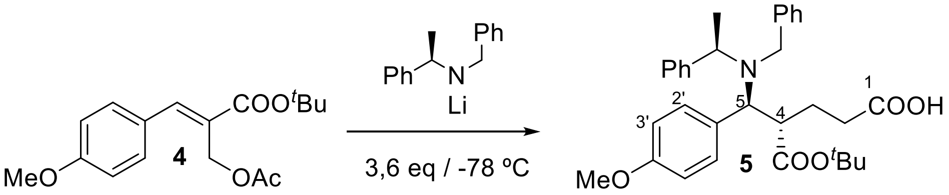

On a (*R*)-(α-methylbenzyl)benzylamine solution, 1.41 g (6.69 mmol) in 5 mL of dry THF, under Ar atmosphere, in a heart flask, 3.94 mL (6.32 mmol) of *n*-BuLi were carefully added (CAS: 109-72-8, 1.6 M commercial solution in hexanes) at -78 ºC. The mixture was stirred for 15 minutes. Then, it was transferred to an ice bath for another 15 minutes. After this time, the reaction mixture was cooled back to -78 °C. In parallel, 0.569 g of **4** (1.58 mmol) was dissolved in 5 mL of dry THF and cooled to -78 °C under Ar atmosphere. The solution from the heart flask was then added via "cannula" onto the dissolved substrate. The temperature was maintained at -78 ° C and it was allowed to stir for an hour. Subsequently, 3 mL of saturated ammonium chloride were added and the mixture was allowed to stir until it reached room temperature. THF was evaporated under reduced pressure. The reaction mixture was extracted with EtOAc and washed with 10% aqueous citric acid, H_2O and saturated aqueous NaCl. The organic phase was dried over anhydrous Na_2SO_4, filtered and the solvent was evaporated, obtaining 0.835 g, which were chromatographed on flash silica and eluted with hexane/EtOAc 9:1 to obtain 0.563 g of **5** (59%).

5: (4*S*,5*S*)-5-(Benzyl ((*R*)-1-phenylethyl) amino)-4-(*tert*-butoxycarbonyl)-5-(4-methoxyphenyl)-pentanoic acid.

$[\alpha]_D^{20}$ = -62.3 (c 1.24, $CHCl_3$).

^{1}H NMR (200 MHz, $CDCl_3$) δ (ppm)*: 7.54-7.16 (m, 12H, ArH), 6.98 (d, *J* = 8.3 Hz, 2H, H-2’), 4.14 (q, *J* = 6.3 Hz, 1H, C(α)H), 3.98 (AB, J_{AB} = 13.7 Hz, 1H, NCH_BHPh), 3.80 (m, 1H, H-5), 3.83 (s, 3H, OMe), 3.57 (AB, J_{AB} = 13.7 Hz, 1H, NCH_AHPh), 3.04 (td, 1H, *J* = 10.8 Hz and 3.0 Hz, H-4), 2.92-2.60 (m, 1H, H-3_A), 2.20-2.10 (m, 2H, H-2), 1.61-1.58 (m, 1H, H-3_B), 1.04 (s, 9H, COO*t*Bu), 0.98 (d, *J* = 6.7 Hz, 3H, C(α)H).

^{13}C NMR (50 MHz, $CDCl_3$) δ (ppm)*: 179.10 (C, COOH), 173.31 (C, COO*t*Bu), 158.82 (C, C-4’), 144.40 (C, C_{ipso}), 140.21 (C, C_{ipso} CH_2Ph), 131.43 (C, C-1’), 130.62 (CH, Ar), 129.26 (CH, Ar), 128.30 (CH, Ar), 128.09 (CH, Ar), 126.96 (CH, Ar), 126.73 (CH, C-2’), 113.17 (CH, C-3’), 80.29 (C,

C(CH3)3), 61.95 (CH, C-5), 55.79 (CH, C(α)), 55.26 (CH_3, OCH_3), 50.60 (CH_2, NCH_2Ph), 49.13 (CH, C-4), 31.51 (CH_2, C-2), 27.47 ((CH_3, C($\underline{C}H_3$)$_3$), 25.47 (CH_2, C-3), 14.34 (CH_3, C(α)Me).

IRυ$_{max}$ (film) (cm^{-1}): 700, 733, 1030, 1150, 1179, 1207, 1250, 1304, 1368, 1452, 1512, 1609, 1724, 2849, 2928, 2974.

E.M.AR., FAB, calcd for $C_{32}H_{40}NO_5$ [M+H]: 518.2901 Found: 518.2896 Δ = -0.77 ppm.

*Compounds **6**-**8** keep same numeration than **5** in the spectroscopic assignation for comparative purposes.

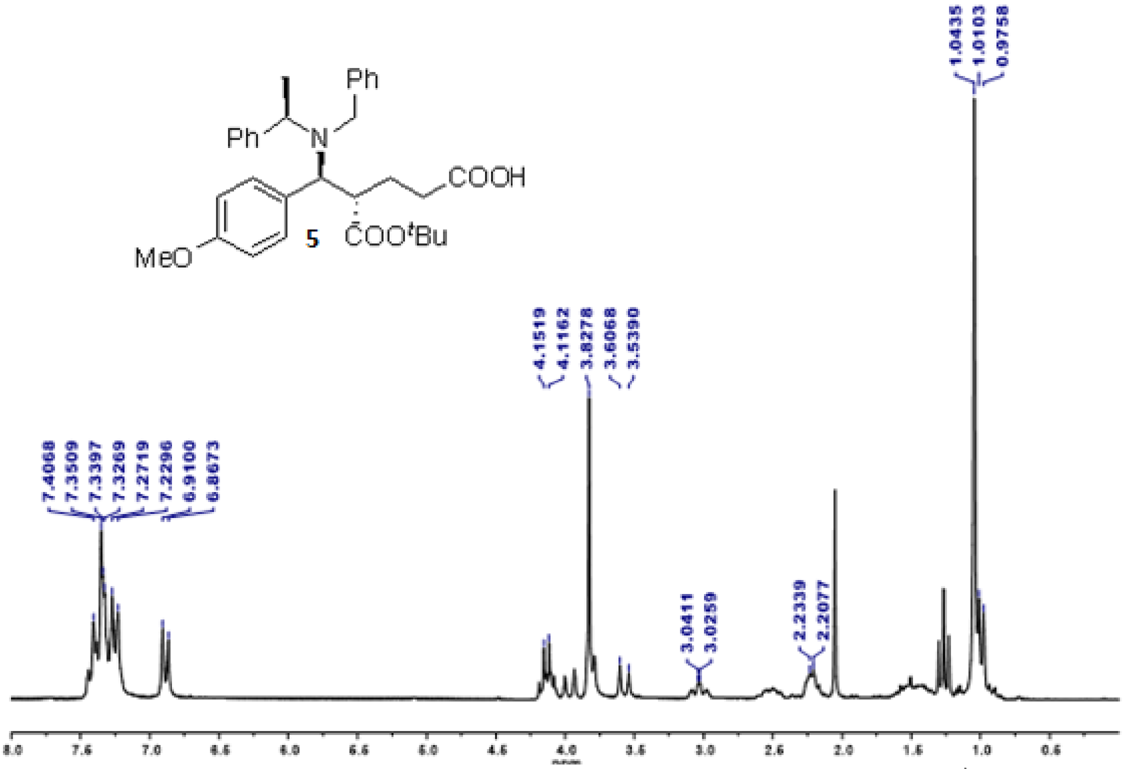

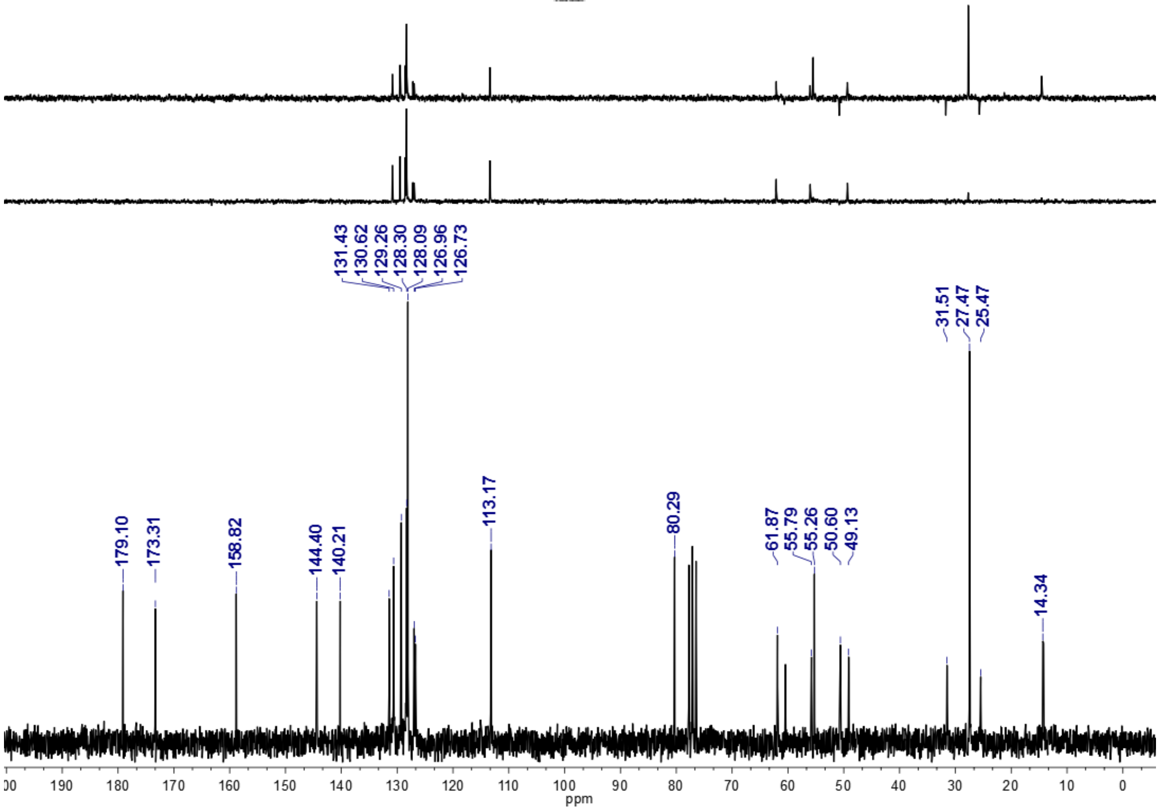

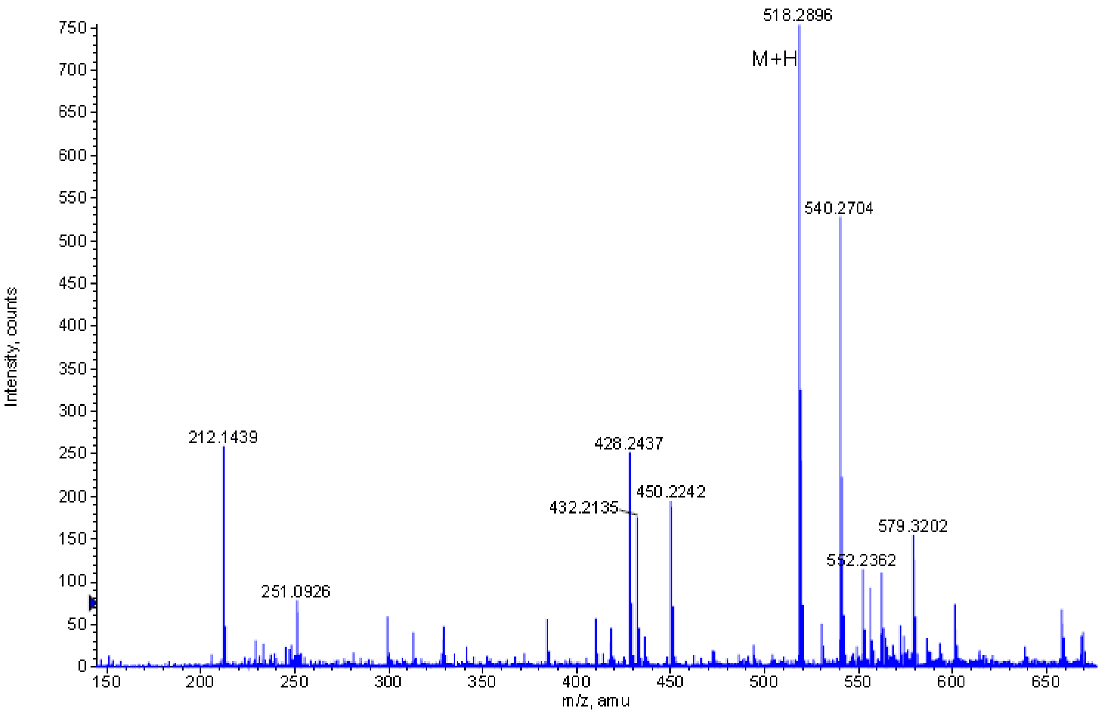

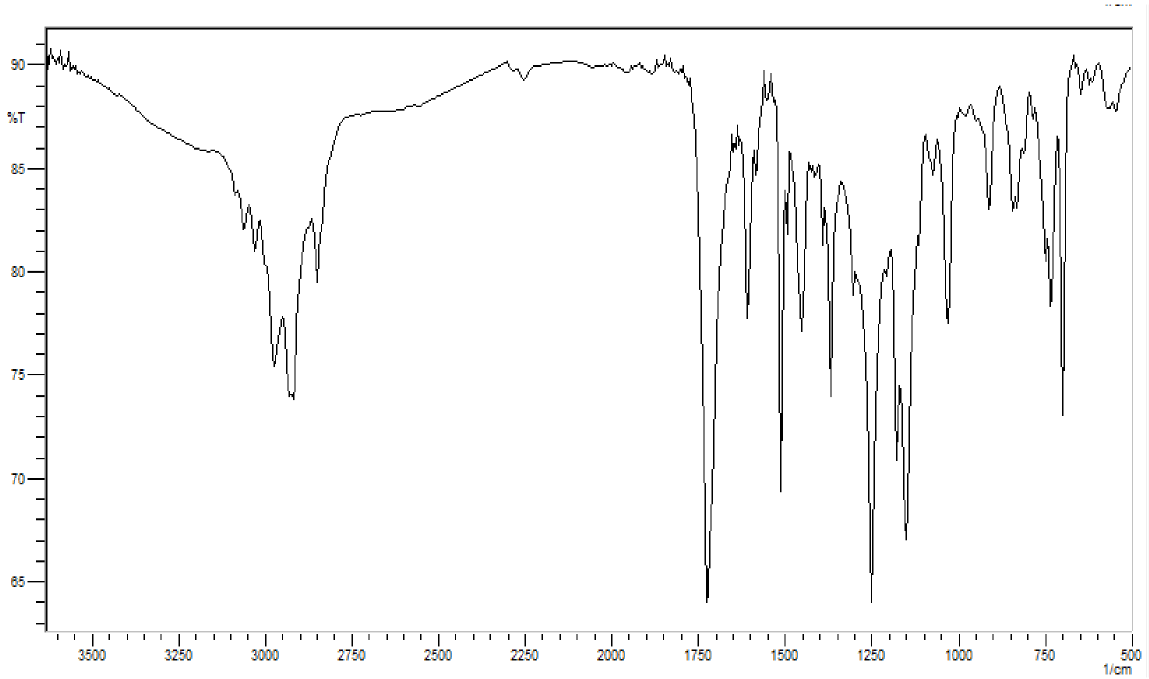

4. Reaction of δ-amino acid 5 with trimethylsilyldiazomethane.

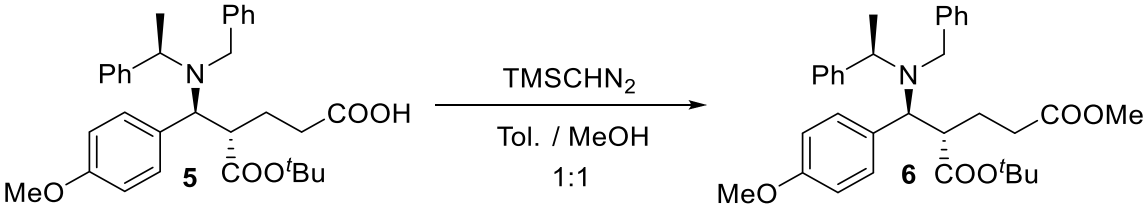

To a solution of **5** (57,6 mg; 0,111 mmol) in 2 mL of toluene-methanol 1:1, 90 µL (0,183 mmol) of trimethylsilyldiazomethane (CAS: 18107-18-1, commercial solution 2M in hexanes, yellow) were added. The mixture was allowed to stir at room temperature for 30 minutes. Then, the crude was evaporated and the desired product was obtained, 59 mg of **6** (100%).

6: 1-(*tert*-butyl) 5-methyl (*S*)-2-((*S*)-(benzyl((*R*)-1-phenylethyl)amino)(4-methoxyphenyl)methyl)glutarate

[α]$_D^{20}$ = - 54.3 (c 1.02, $CHCl_3$).

^{1}H NMR (200 MHz, $CDCl_3$) δ (ppm): 7.58-7.02 (m, 12H, ArH), 6.89 (d, *J* = 8.3 Hz, 2H, H-3'), 4.14 (q, *J* = 6.5 Hz, 1H, C(α)H), 3.97 (AB, J_{AB} = 13.7 Hz, 1H, NCH_BHPh), 3.83 (s, 3H, OMe), 3.82 (d, 1H, *J* = 10.8 Hz, H-5),3.68 (s, 3H, COOMe), 3.58 (AB, J_{AB} = 13.7 Hz, 1H, NCH_AHPh), 2.98 (td, *J* = 11.0, 3.0 Hz, 1H, H-4), 2.71-2.43 (m, 1H, H-3_A), 2.23-2.08 (m, 2H, H-2), 1.76-1.48 (m, 1H, H-3_B), 1.02 (s, 9H, *t*Bu), 0.97 (s, 3H, C(α)H).

^{13}C NMR (50 MHz, $CDCl_3$) δ (ppm): 173.51 (C, COOMe), 173.22 (C, COO*t*Bu), 158.83 (C, C-4'), 144.40 (C, C_{ipso} CHPh), 140.23 (C, C_{ipso} CH_2Ph), 131.50 (C, C-1'), 130.60 - 126.60 (CHx12, Ar), 113.15 (CH, C-2'), 80.10, (C, $\underline{C}(CH_3)_3$) 61.85 (CH, C-5), 55.74 (CH, C(α)), 55.24 (CH_3, OCH_3), 51.45 (CH_3, COOMe), 50.59 (CH_2, NCH_2Ph), 49.18 (CH, C-4), 31.54 (CH_2, C-2), 27.49 (CH_3, $C(\underline{C}H_3)_3$), 25.72 (CH_2, C-3), 14.34 (CH_3, C(α)Me).

IRυ_{max} (film) (cm^{-1}): 700.16, 1031.92, 1151.50, 1176.58, 1251.80, 1367.53, 1454.33, 1512.19, 1606.70, 1697.36, 1714.72, 1728.22, 2927.94

E.M.AR., FAB, calcld for $C_{33}H_{41}O_5N$ [M+Na]: 554.2877. Found: 554.2886. Δ =1,63 ppm.

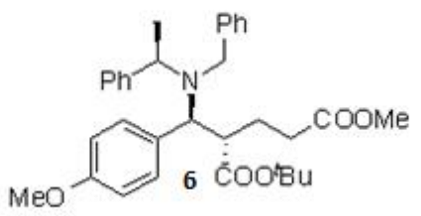

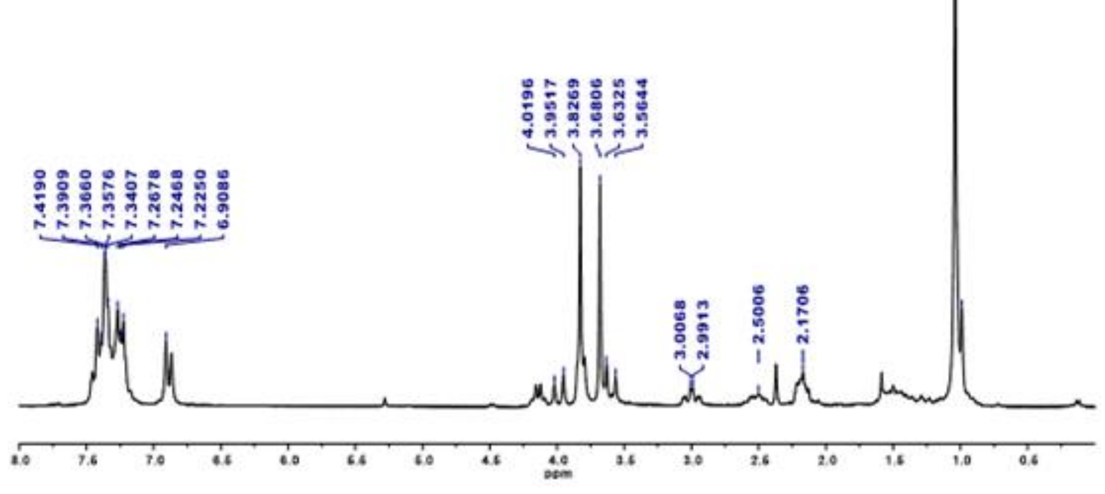

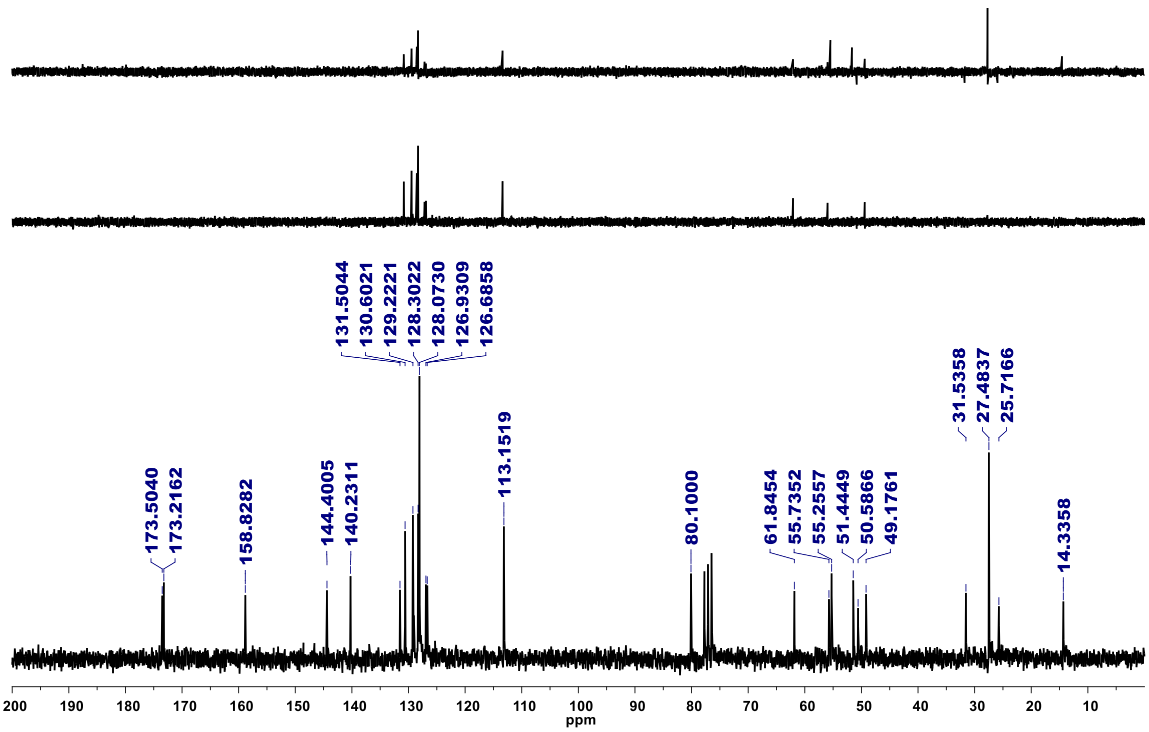

173.5040
173.2162
158.8282
144.4005
140.2311
131.5044
130.6021
129.2221
128.3022
128.0730
126.9309
126.6858
113.1519
80.1000
61.8454
55.7352
55.2557
51.4449
50.5866
49.1761
31.5358
27.4837
25.7166
14.3358
ppm

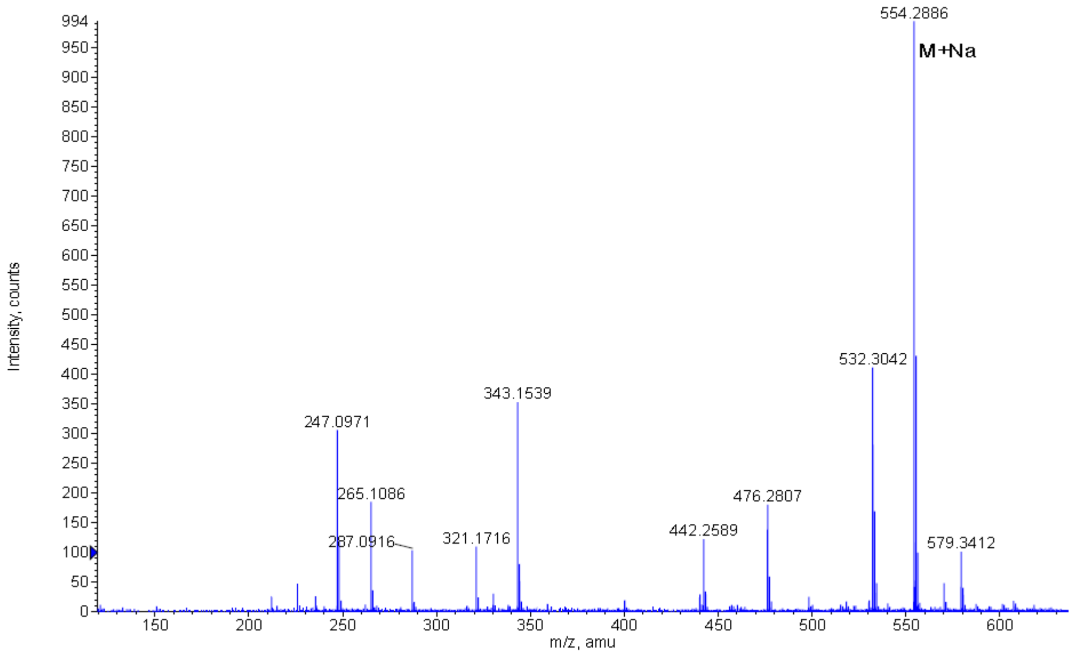

554.2886
M+Na
532.3042
343.1539
247.0971
265.1086
287.0916
321.1716
442.2589
476.2807
579.3412
Intensity, counts
m/z, amu

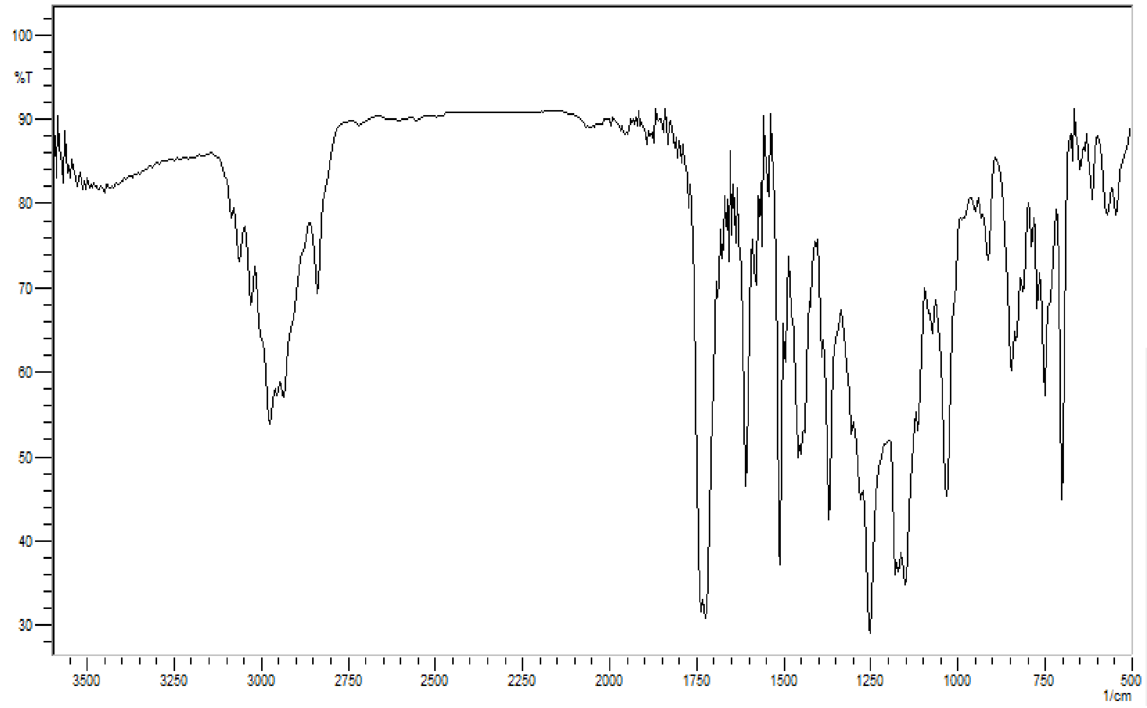

5. Obtaining the *β*-amino acid 7.

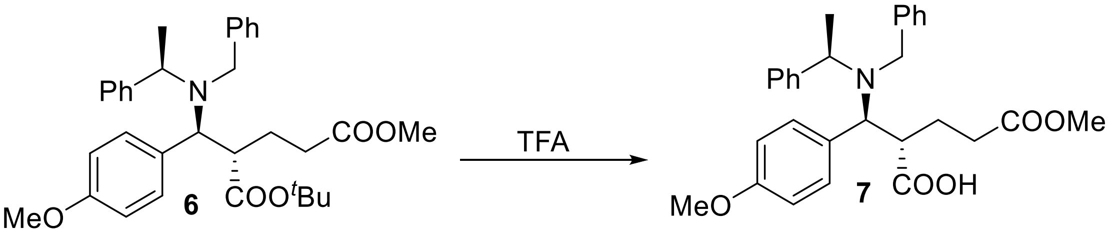

0.9 mL of trifluoroacetic acid (CAS: 76-05-1, colourless liquid) were added to 50 mg of **6** (0.094 mmol) and the mixture was allowed to stir at room temperature for 4 h. After this time the volatile compounds were evaporate. The crude was dissolved in EtOAc and washed with 5% aqueous $NaHCO_3$ and saturated aqueous NaCl. The organic phase was dried over anhydrous Na_2SO_4, filtered and the solvent was evaporated, obtaining 42 mg of **7** (95%).

7: (*S*)-2-((*S*)-(benzyl((*R*)-1-phenylethyl)amino)(4-methoxyphenyl)methyl)-5-methoxy-5-oxopentanoic acid.

[α]$_D$^{20} = + 17.7 (c 1.27, $CHCl_3$).

^{1}H NMR (200 MHz, $CDCl_3$) δ (ppm): 7.58 - 7.02 (m, 12H, ArH), 6.78 (d, J = 8.3 Hz, 2H, H-2'), 4.12 (q, J = 6.8 Hz, 1H, C(α)H), 3.80 (AB, J_{AB} = 13.7 Hz, 1H, NCH_BHPh), 3.76 (s, 3H, OMe), 3.62 (s, 3H, COOMe), 3.61 (AB, J_{AB} = 13.7 Hz, 1H, NCH_AHPh), 2.62 (td, J = 11.0, 3.0 Hz, 1H), 2.30-2.20 (m, 2H, H-2), 1.76-1.48 (m, 2H), 1.37 (d, J = 6.8 Hz, 3H, C(α)H).

^{13}C NMR (50 MHz, $CDCl_3$) δ (ppm): 176.22 (C, COOH), 174.13 (C, COOMe), 159.77 (C, C-4'), 144.61 (C, C_{ipso} CH_2Ph), 137.72 (C, C_{ipso}), 131.05 (C, C1'), 129.00-127.79 (CHx12, Ar), 114.28 (CH, C-2'), 62.55 (CH, C-5), 57.16 (CH, C(α)), 55.43 (CH_3, OCH_3), 51.69(CH_3, COOMe), 50.80 (CH_2, NCH_2Ph), 44.92 (CH, C-4), 32.15 (CH_2, C-2), 24.56 (CH_2, C-3), 14.42 (CH_3, C(α)Me).

E.M.AR., FAB, calcld for $C_{29}H_{34}NO_5$ [M+H]: 476,2437. Found: 476.2431. Δ =1.03 ppm.

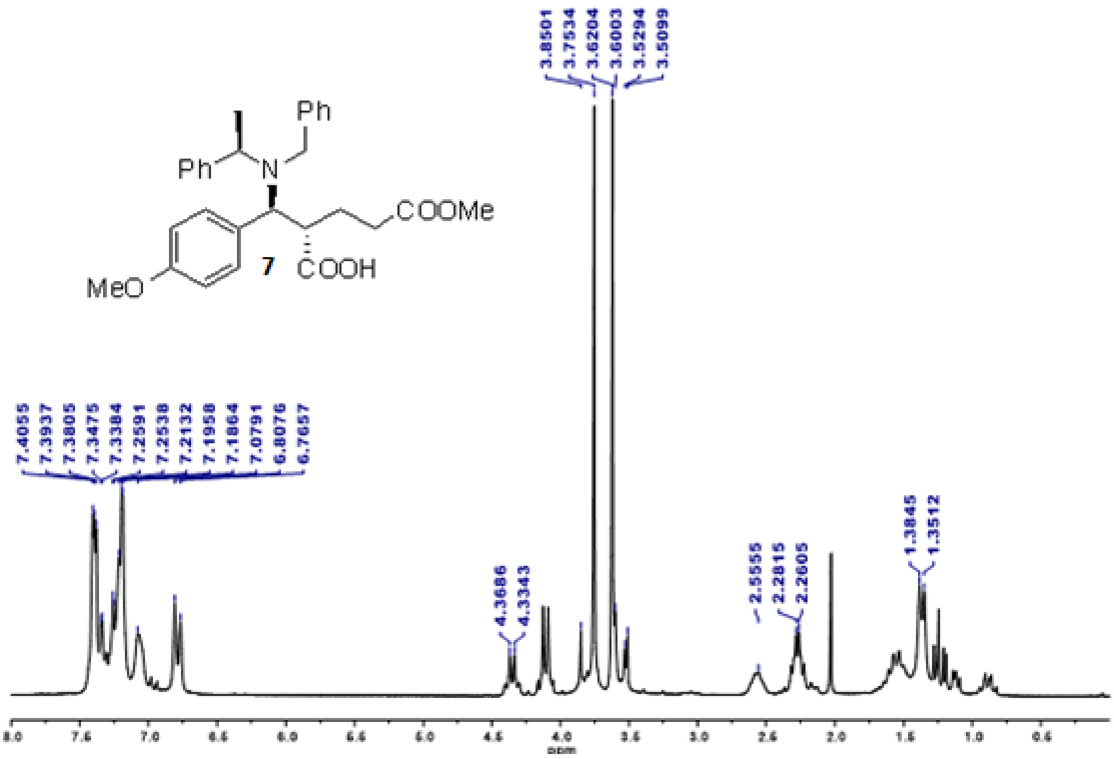
Ph
Ph
N
COOMe
MeO
7
COOH
3.8501
3.7534
3.6204
3.6003
3.5294
3.5099
7.4055
7.3937
7.3805
7.3475
7.3384
7.2591
7.2538
7.2132
7.1958
7.1864
7.0791
6.8076
6.7657
4.3686
4.3343
2.5555
2.2815
2.2605
1.3845
1.3512
ppm

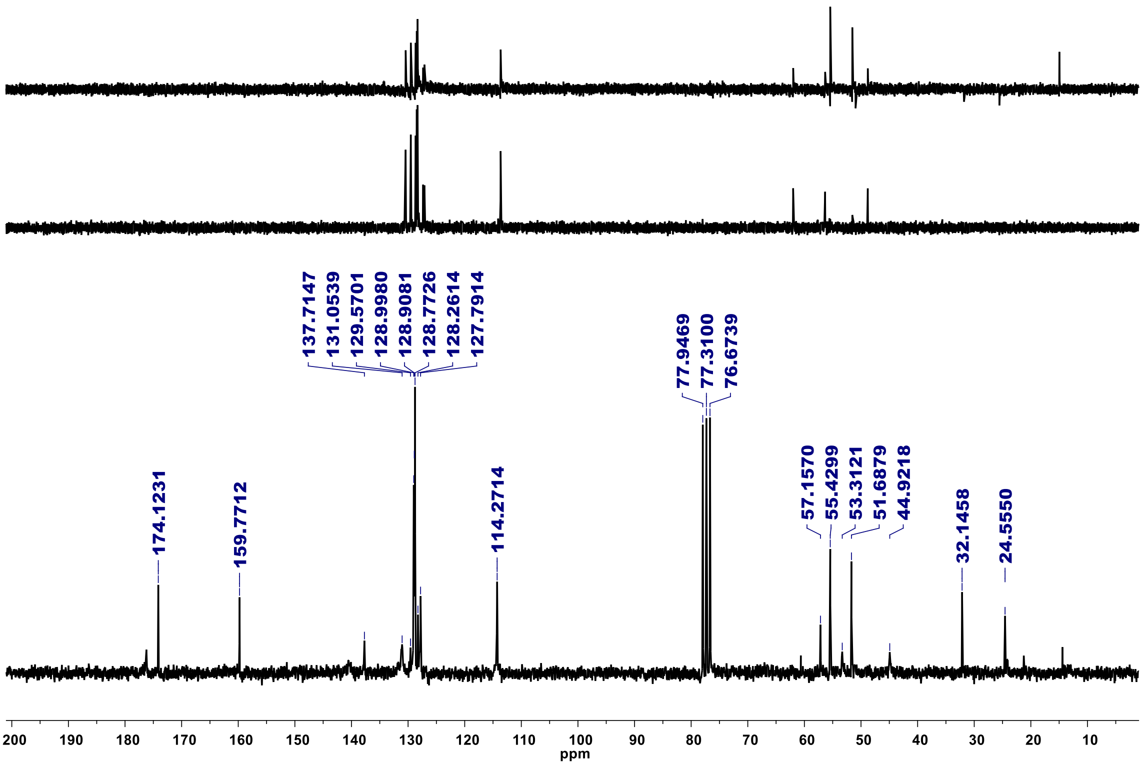
137.7147
131.0539
129.5701
128.9980
128.9081
128.7726
128.2614
127.7914
77.9469
77.3100
76.6739
174.1231
159.7712
114.2714
57.1570
55.4299
53.3121
51.6879
44.9218
32.1458
24.5550
200
190
180
170
160
150
140
130
120
110
100
90
80
70
60
50
40
30
20
10
ppm

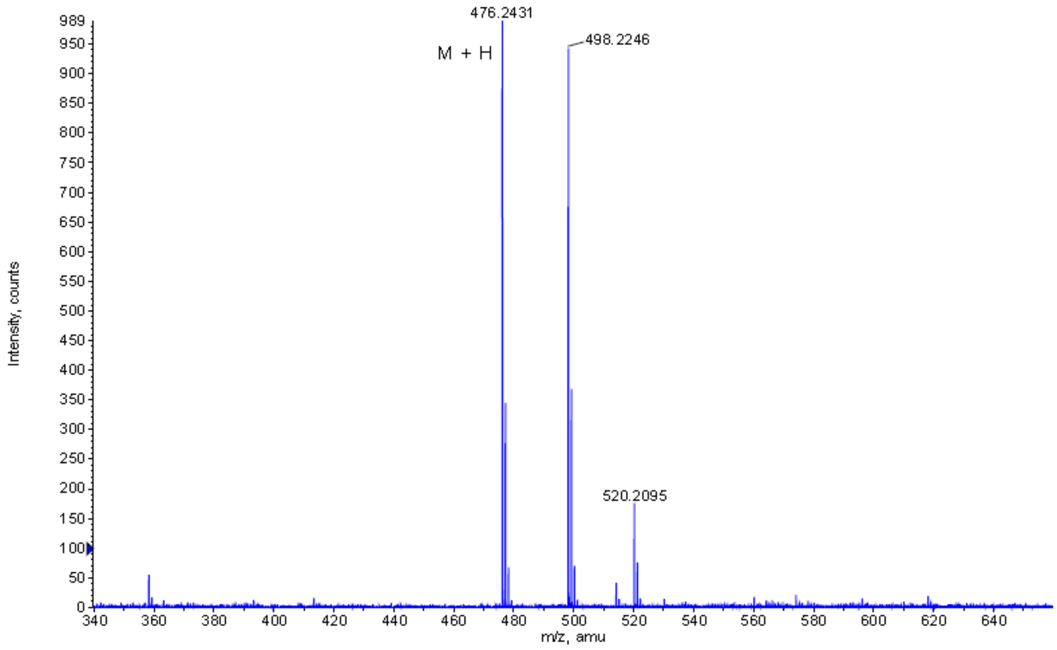

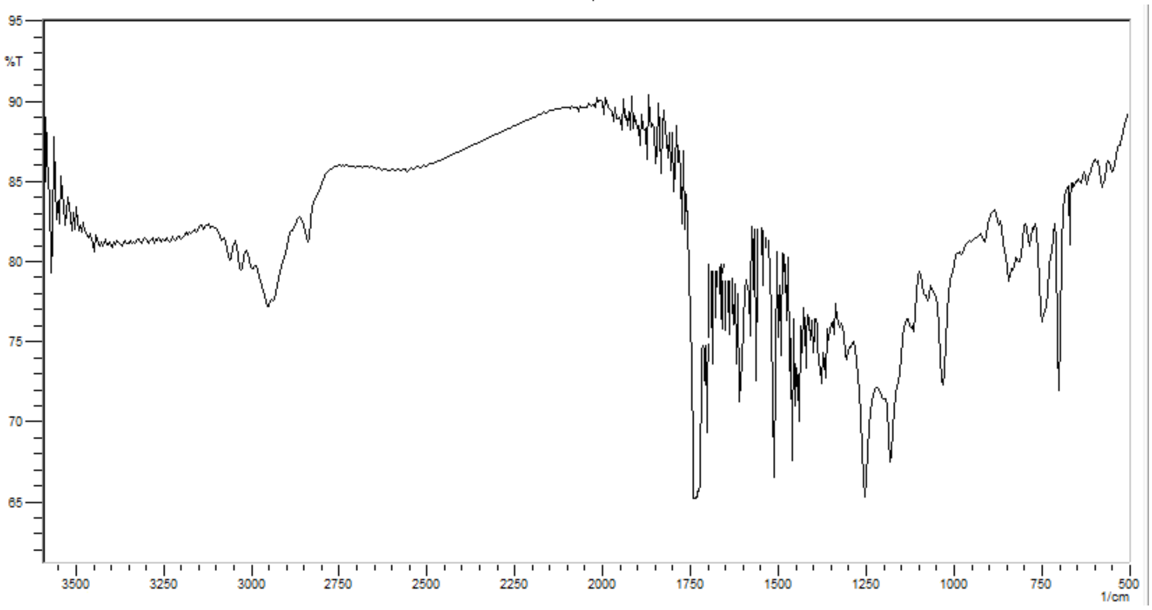

6. Chemoselective debenzylation of 7.

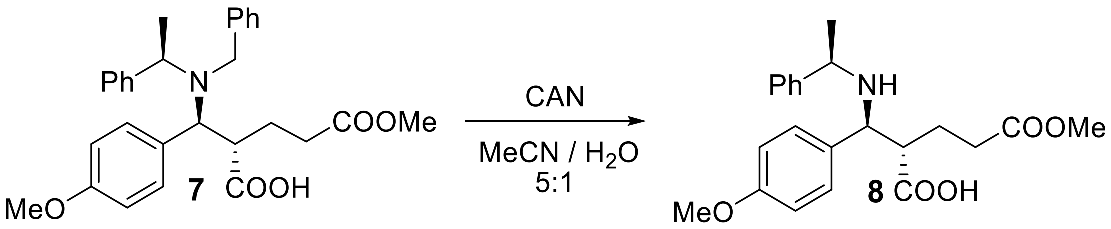

To a solution of **7** (58.3 mg; 0.124 mmol) in 12 mL of acetonitrile water 5:1, 211 mg (0.498 mmol) of CAN (CAS: 16774-21-3, orange solid) were added. The mixture was kept under stirring at room temperature under Ar atmosphere for 15 minutes. After this time, 5 mL of saturated aqueous $NaHCO_3$ were added and the mixture was left to stir for another 15 minutes. The reaction mixture is extracted with DCM. The organic phase was dried over anhydrous Na_2SO_4, filtered and the solvent was evaporated, obtaining 62 mg, which were chromatographed on flash silica. The desired product was eluted with hexane/EtOAc 7:3, 40 mg of **8** (84.0%)

8: (*S*)-5-methoxy-2-((*S*)-(4-methoxyphenyl)(((*R*)-1-phenylethyl)amino)methyl)-5-oxopentanoic acid.

[α]$_D$^{20} = + 19.5 (c 1.12, $CHCl_3$).

^{1}H NMR (200 MHz, $CDCl_3$) δ (ppm): 7.41-7.26 (m, 5H, ArH), 7.04 (d, *J* = 4 Hz, 2H, H2'), 6.80 (d, *J* = 8.4 Hz, 2H, H-3'), 4.03 (q, *J* = 6.8 Hz, 1H, C(α)H), 3.83 (AB, J_{AB} = 13.7 Hz, 1H, NCH_BHPh), 3.76 (s, 3H, OMe), 3.62 (s, 3H COOMe), 3.58 (AB, J_{AB} = 13.7 Hz, 1H, NCH_AHPh), 2.84 (td, *J* = 11.0 and 3.0 Hz, 1H), H-4), 2.43 (t, *J* = 7.6, 2H, H-2), 1.82-1.65 (m, 1H), 1.51 (d, *J* = 6.8 Hz, 3H, C(α)H), 1.42-1.25 (m, 2H).

^{13}C NMR (50 MHz, $CDCl_3$) δ (ppm): 177.63 (C, COOH), 173.82 (C, COOMe), 159.14 (C, C-4'), 143.46 (C, C_{ipso} CH_2Ph), 130.75 (C, C_{ipso}), 128.93 (C, C-1'), 128.54-126.96 (CHx9, Ar), 113.88 (CH, C-3'), 61.04 (CH, C-5), 55.31 (CH, C(α)), 55.19 (CH_3, OCH_3), 51.56 (CH_3, COOMe), 51.03 (CH_2, NCH_2Ph), 32.54 (CH, C-4), 24.58 (CH_2, C-2), 20.56 (CH_2, C-3), 14.21 (CH_3, C(α)Me).

IRυ_{max} (film) (cm^{-1}): 702, 733, 831, 1034, 1180, 1211, 1252, 1306, 1400, 1508, 1611, 1701, 1719, 1730, 2837, 2953.

E.M.AR., FAB, calcld for $C_{22}H_{27}NO_5Na$ [M+Na]: 408.1781. Found: 408.1777. Δ = -1.09 ppm.

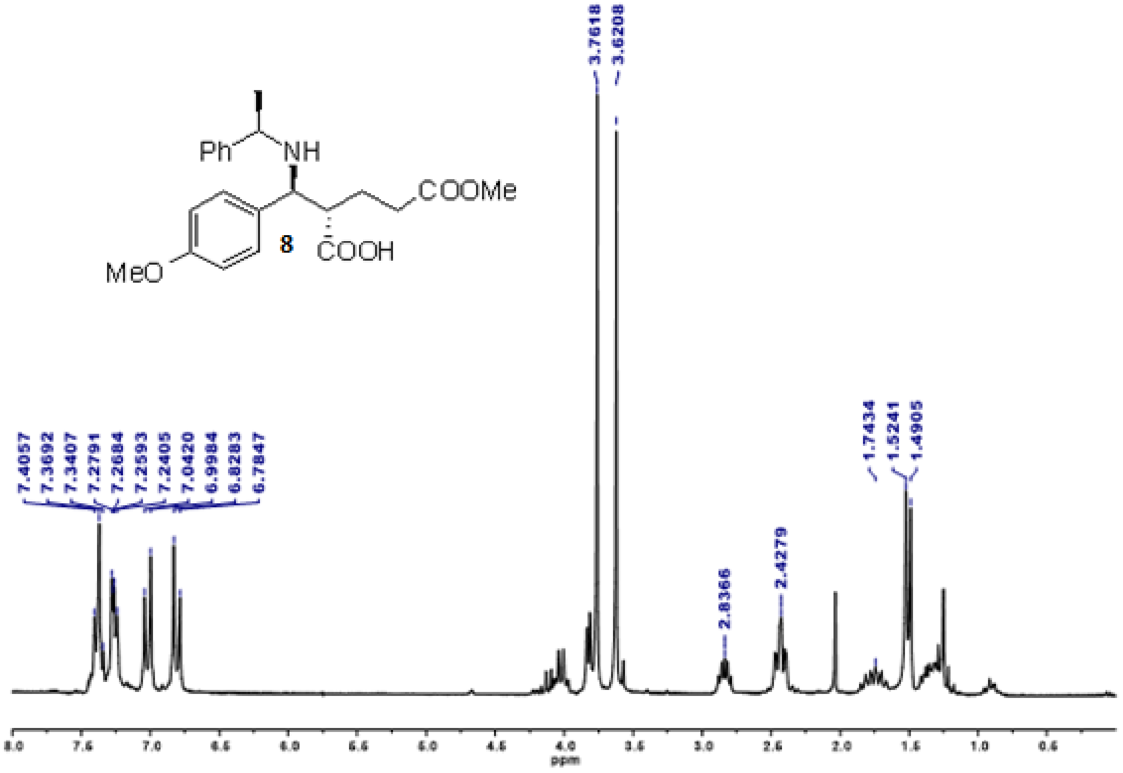

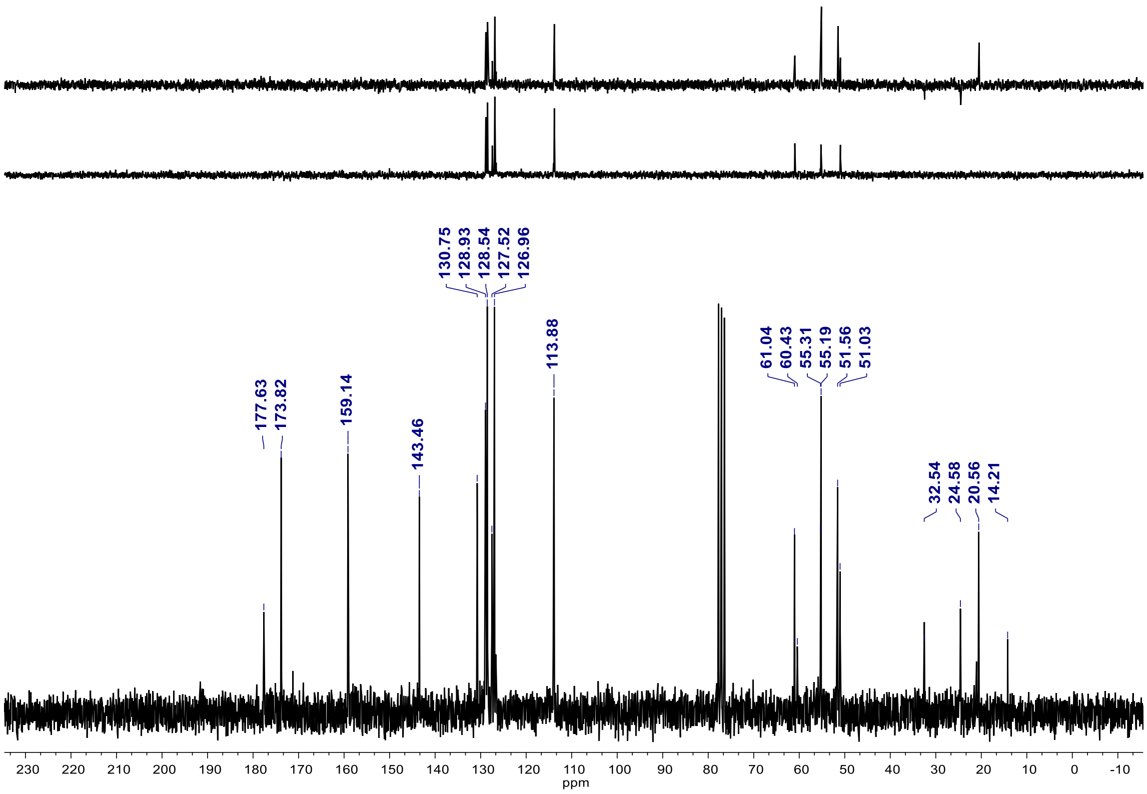
177.63
173.82
159.14
143.46
130.75
128.93
128.54
127.52
126.96
113.88
61.04
60.43
55.31
55.19
51.56
51.03
32.54
24.58
20.56
14.21
230 220 210 200 190 180 170 160 150 140 130 120 110 100 90 80 70 60 50 40 30 20 10 0 -10
ppm

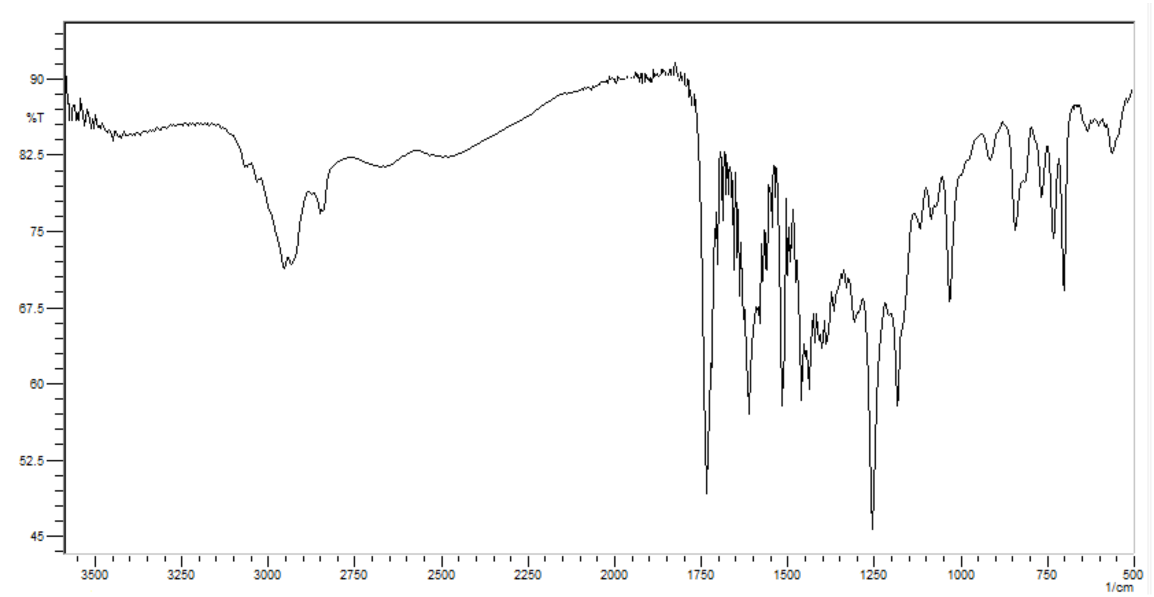

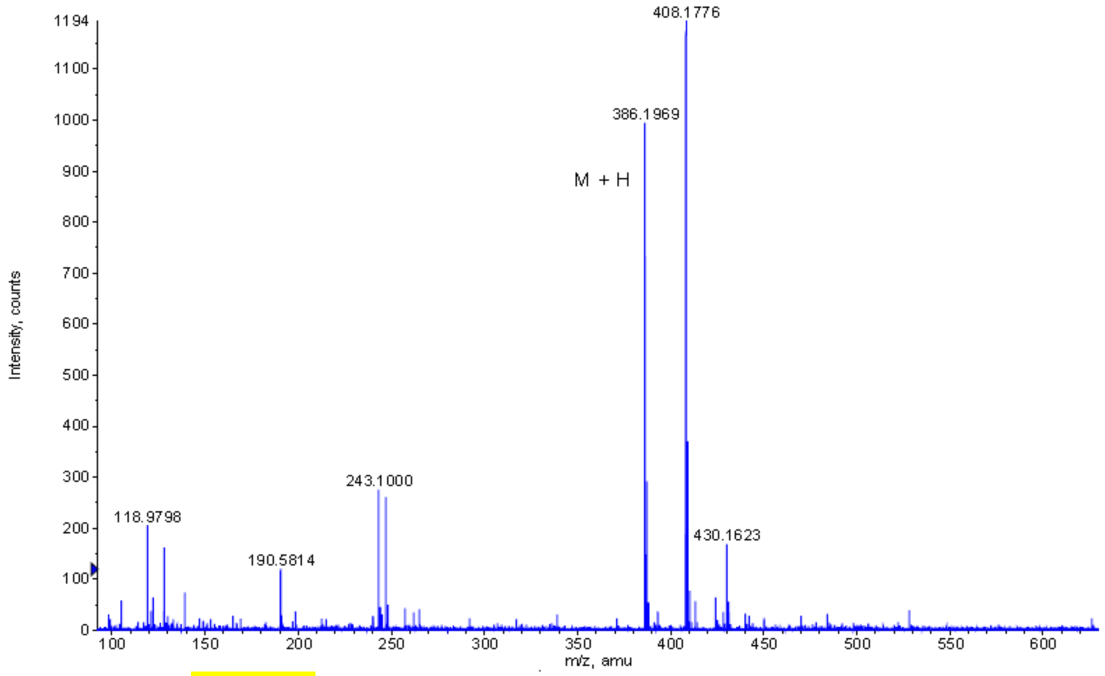

7. Obtaning *β*-lactam 1.

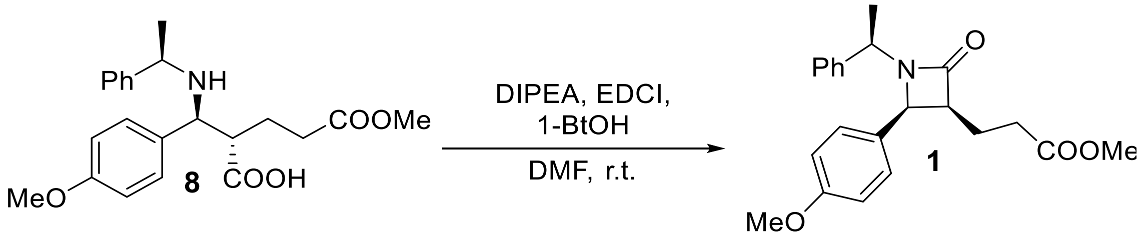

40 mg (0.109 mmol) of **8** were dissolved in 20 mL of DMF. Then, 79 mg (0.24 mmol) of EDCI (CAS: 25952-53-8, white powder), 55.8 mg (0.24 mmol) of hydroxybenzotriazole hydrate (CAS: 123333-53-9, white powder) and 64 µL (0.24 mmol) of DIPEA (CAS: 7087-68-5, colourless liquid) were added. Stirring was maintained for 16 h, sealing the balloon with a $CaCl_2$ tube. The crude was dissolved in EtOAc and washed with small portions of H_2O and saturated aqueous NaCl. The organic phase was dried over anhydrous Na_2SO_4, filtered and the solvent was

evaporated, obtaining 38 mg, which were chromatographed on "flash" silica. The desired product was eluted with hexane/EtOAc 9:1, 33mg of **1** (86.7%) were obtained.

1: (3*S*,4*S*)-*N*-[(*R*)-α-methylbenzylamine]-4-*p*-methoxyphenyl-3-methoxycanbonylethyl-β-lactam.

[α]$_D$^{20} = + 103.5 (c 1.19, $CHCl_3$).

^{1}H NMR (200 MHz, $CDCl_3$) δ (ppm): 6.90-6.79 (m, 5H, ArH), 6.75 (d, *J* = 8.7 Hz, 2H, H-2'), 6.49 (d, *J* = 8.7 Hz, 2H, H-3'), 4.57 (q, *J* = 7.2 Hz, 1H, C(α)), 4.12 (d, *J* = 5.6 Hz, 1H, H-4), 3.36 (s, 3H, COOMe), 3.15 (s, 3H, OMe), 2.83 (td, *J* = 8.3, 5.6 Hz, 1H, H-3), 1.82-1.72 (m, 2H, H-2''), 1.45-1.31 (m, 2H, H-1''), 1.00 (d, *J* = 7.2 Hz, 3H, C(α)Me).

^{13}C NMR (50 MHz, $CDCl_3$) δ 173.24 (C, COOMe), 170.548(C, C-2), 159.71 (C, C-4'), 140.56 (C, C_{ipso} CHPh), 129.05 (CH, Ar), 128.81 (CH, Ar), 128.42 (CH, Ar), 127.86 (CH, Ar), 127.40 (CH, Ar), 113.77 (CH, C-2'), 57.56 (CH, C(α)), 55.36 (CH_3, OCH_3), 53.48 (CH, C-4),52.69 (CH, C-3), 51.63 (CH_3, $COOCH_3$), 31.30 (CH_2, C-2''), 21.08 (CH_2, C-1''), 19.65 (CH_3, C(α)Me).

IRυ_{max} (film) (cm^{-1}): 703, 733, 1032, 1182, 1256, 1292, 1306, 1356, 1389, 1400, 1516, 1611, 1734, 2934, 2953.

E.M.AR., FAB, calcld para $C_{22}H_{25}NO_4Na$ [M+Na]: 390.1676. Found: 390.1679. Δ = 0,82 ppm.

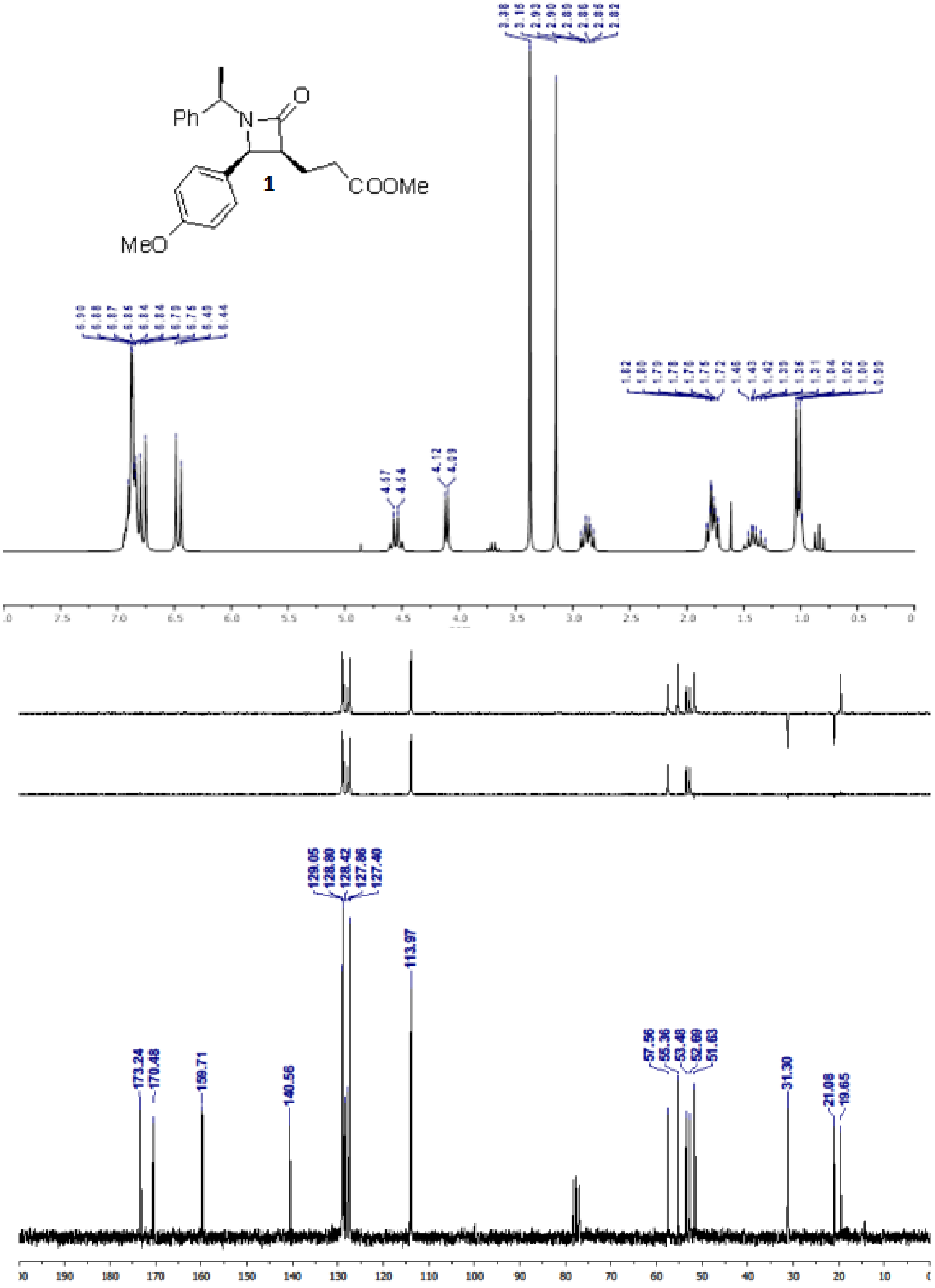
Ph
N
O
COOMe
MeO
1
4.57
4.54
4.12
4.09
173.24
170.48
159.71
140.56
129.05
128.80
128.42
127.86
127.40
113.97
57.56
55.36
53.48
52.69
51.63
31.30
21.08
19.65

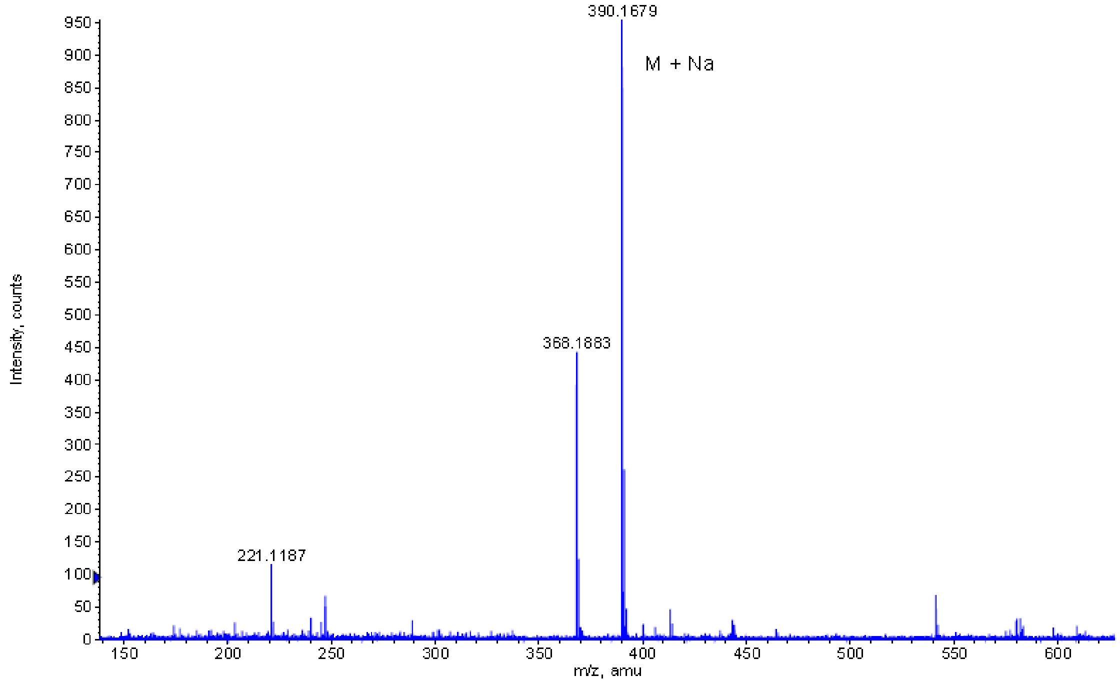
390.1679
M + Na
368.1883
221.1187
Intensity, counts
m/z, amu
950
900
850
800
750
700
650
600
550
500
450
400
350
300
250
200
150
100
50
0
150
200
250
300
350
400
450
500
550
600

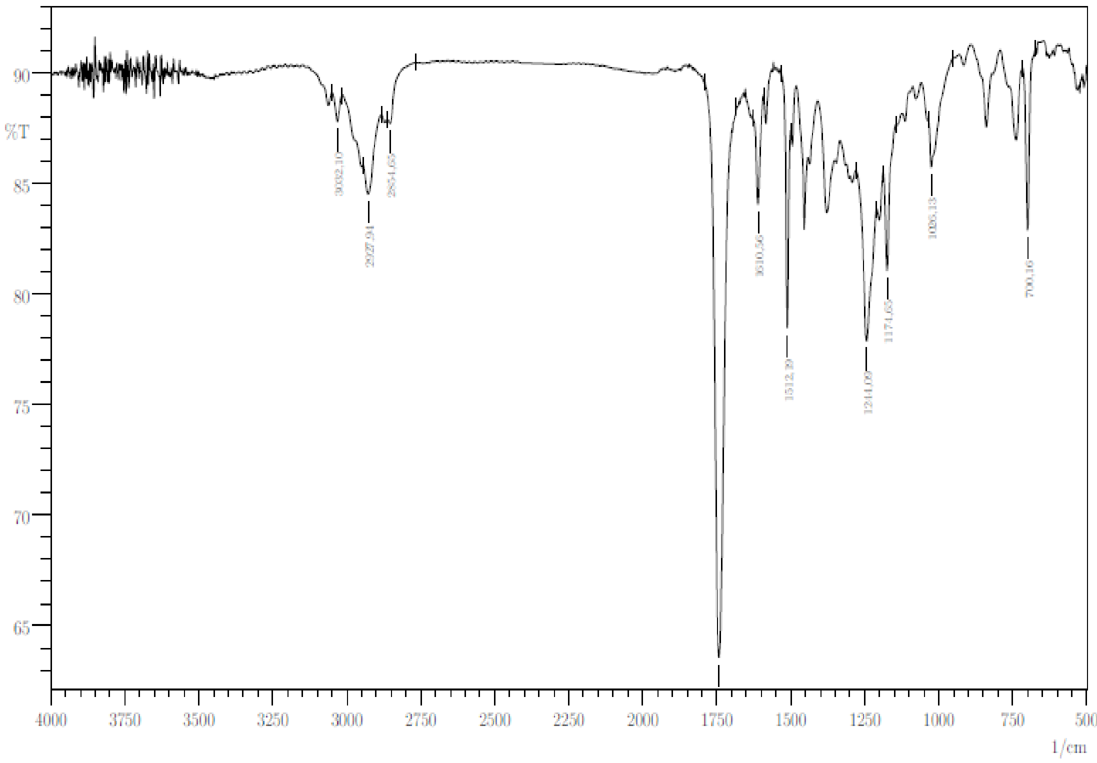
%T
90
85
80
75
70
65
3032.10
2927.94
1610.56
1244.09
1171.65
1026.13
700.16
4000
3750
3500
3250
3000
2750
2500
2250
2000
1750
1500
1250
1000
750
500
1/cm
